# Genotypic Regulation of Aflatoxin Accumulation but Not *Aspergillus* Fungal Growth upon Post-Harvest Infection of Peanut (*Arachis hypogaea* L.) Seeds

**DOI:** 10.3390/toxins9070218

**Published:** 2017-07-12

**Authors:** Walid Ahmed Korani, Ye Chu, Corley Holbrook, Josh Clevenger, Peggy Ozias-Akins

**Affiliations:** 1 Institute of Plant Breeding, Genetics and Genomics, University of Georgia, Tifton, GA 31793, USA; korani@uga.edu (W.A.K.); ye.chu.test@gmail.com (Y.C.); jclev@uga.edu (J.C.); 2 The United States Department of Agriculture—Agricultural Research Service, Crop Genetics and Breeding Research Unit, Tifton, GA 31793, USA; corley.holbrook@ars.usda.gov

**Keywords:** aflatoxin, *Aspergillus flavus*, peanut, GFP, SICIA

## Abstract

Aflatoxin contamination is a major economic and food safety concern for the peanut industry that largely could be mitigated by genetic resistance. To screen peanut for aflatoxin resistance, ten genotypes were infected with a green fluorescent protein (GFP)—expressing *Aspergillus flavus* strain. Percentages of fungal infected area and fungal GFP signal intensity were documented by visual ratings every 8 h for 72 h after inoculation. Significant genotypic differences in fungal growth rates were documented by repeated measures and area under the disease progress curve (AUDPC) analyses. SICIA (Seed Infection Coverage and Intensity Analyzer), an image processing software, was developed to digitize fungal GFP signals. Data from SICIA image analysis confirmed visual rating results validating its utility for quantifying fungal growth. Among the tested peanut genotypes, NC 3033 and GT-C20 supported the lowest and highest fungal growth on the surface of peanut seeds, respectively. Although differential fungal growth was observed on the surface of peanut seeds, total fungal growth in the seeds was not significantly different across genotypes based on a fluorometric GFP assay. Significant differences in aflatoxin B levels were detected across peanut genotypes. ICG 1471 had the lowest aflatoxin level whereas Florida-07 had the highest. Two-year aflatoxin tests under simulated late-season drought also showed that ICG 1471 had reduced aflatoxin production under pre-harvest field conditions. These results suggest that all peanut genotypes support *A. flavus* fungal growth yet differentially influence aflatoxin production.

## 1. Introduction

*Aspergillus flavus* is an opportunistic pathogen with a wide host range including peanut, corn, wheat, barley, rice, tree nuts, and cotton seeds [1,2,3,4,5,6]. Peanut is one of the most susceptible crops to *A. flavus* and *A. parasiticus* infection either in the field (pre-harvest) or during storage (post-harvest) [7,8]. *A. flavus* and *A. parasiticus* produce aflatoxins as secondary metabolites under conducive environmental conditions. Aflatoxins cause toxicosis, cancer, and immunosuppressive diseases in animals including humans [9,10] and alfatoxin B1 level is highly regulated worldwide [11]. Aflatoxin contamination incurs an average $20 million annual cost to the United States peanut industry [12].

*Aspergillus* spp. conidia and sclerotia are abundant in the soil and can survive through harsh weather conditions. Since peanut pods develop underground, direct contact of developing peanut pods with fungal mycelium provides the main entry for fungal invasion [13,14]. Peanut pods damaged by insects and nematodes were shown to have elevated levels of aflatoxin contamination [15,16]. Another reported route of fungal infection is through flowers [17]. Heat and drought stress in the field exacerbates aflatoxin contamination [18]. Peanut host genes altered by *A. flavus* contamination were discovered by a genome wide RNA-seq analysis [19]. Disruption of peanut abscisic acid signaling pathway by *A. flavus* invasion was suggested to facilitate aflatoxin accumulation. As for minimizing post-harvest aflatoxin contamination, clean, dry and temperature controlled storage conditions with protection from insect and rodent infestation are recommended [14]. In developing countries, appropriate storage conditions are often inaccessible or unaffordable. 

Development of aflatoxin resistant peanut cultivars has been one of the most challenging goals due to the large variation in pre-harvest aflatoxin contamination. Even aflatoxin resistant lines accumulated widely different levels of aflatoxin when grown in the same or different environments [20]. A recent gene profiling study comparing an aflatoxin resistant line and a susceptible line revealed multiple biological pathways enriched in the resistant line upon *A. flavus* challenge [21]. However, the resistant line accumulated over 20,000 ppb of aflatoxin over the 10-d time frame of the experiment, which even though 1% of the aflatoxin in the susceptible line, far exceeds the United States action level of 20 ppb for human food consumption and is not actually resistant.

To circumvent the high variation in field aflatoxin evaluation of genetic resistance, in vitro inoculation has been used to ensure more uniform fungal infection of seeds. Several wild diploid peanut relatives and interspecific tetraploids were reported to be resistant to aflatoxin based on in vitro inoculation of seeds and analysis 8-d post inoculation [8]. Since complete inhibition of fungal growth or aflatoxin contamination is unlikely, a time course to monitor fungal growth during the disease progression could reveal differential fungal–host interactions among peanut genotypes. Surrogate parameters estimating fungal growth such as β-l-3-glucanase activity previously were used in in vitro studies [22,23] but are destructive assays. Green fluorescent protein (from *Aequorea victoria*) transformed *A. flavus* allows for the non-destructive measurement of fungal growth [24] and AF-70-GFP, a GFP-expressing *A. flavus* strain, was used in cotton to identify resistant cotton lines [2]. Since dissociation of in vitro aflatoxin resistance and pre-harvest aflatoxin contamination was reported previously [25], it is possible that in vitro and field tests were interrogating different aspects of aflatoxin resistance mechanisms in the genotypes examined.

In this study, ten peanut genotypes were selected based on their previously reported resistance to aflatoxin contamination and or drought tolerance. ICGV88145 [26] and ICG 1471 [27,28,29,30] are aflatoxin resistant lines released by the International Crops Research Institute for the Semi-Arid Tropics (ICRISAT) and Senegal, respectively. ICG 1471 also is drought tolerant [31]. GT-C20 is a Chinese cultivar [32] reported to retard *A. flavus* fungal growth and prevent aflatoxin production [33]. C76-16 is a drought tolerant USDA breeding line with field aflatoxin resistance [34]. Tifguard [35], NC 3033 [36], Tifrunner [37] and Florida-07 [38] showed less aflatoxin contamination compared to susceptible control breeding line A72 [39]. Another susceptible breeding line, A69, was selected from the cross NCV-11 x GFA-2 from the USDA peanut breeding program in Tifton, GA. In this study, these ten peanut genotypes were inoculated with AF-70-GFP and evaluated for fungal growth by both visual rating and image analysis as well as for aflatoxin contamination over a 3-d time course. Genotypic differences in aflatoxin production per unit of fungus were documented.

## 2. Results and Discussion

It is known that aflatoxin contamination increases in seeds with low viability and germination rate [40,41]. In this study, instead of using seed sources from storage, all genotypes were grown in a common field and harvested at their respective optimum maturity dates. Sound and mature seeds were selected to reduce the variable of maturity. Germination tests of these freshly harvested seeds showed greater than 98% germination rate (data not shown). Since the presence or absence of testa can affect the level of colonization by *Aspergilli* [42], and tannins [43] and antioxidants [44] have been identified in peanut testa, we adopted a method of surface sterilization to preserve testa integrity. Therefore, peanut kernels were UV sterilized prior to inoculation which protected the intactness of peanut testa and minimized the confounding effect of *A. flavus* or other microbiota persisting from the field. The more extensive testa surface area of Florida-07 seeds, which were approximately twice the size of ICG 1471, remained intact with UV sterilization. 

**Figure 1:**
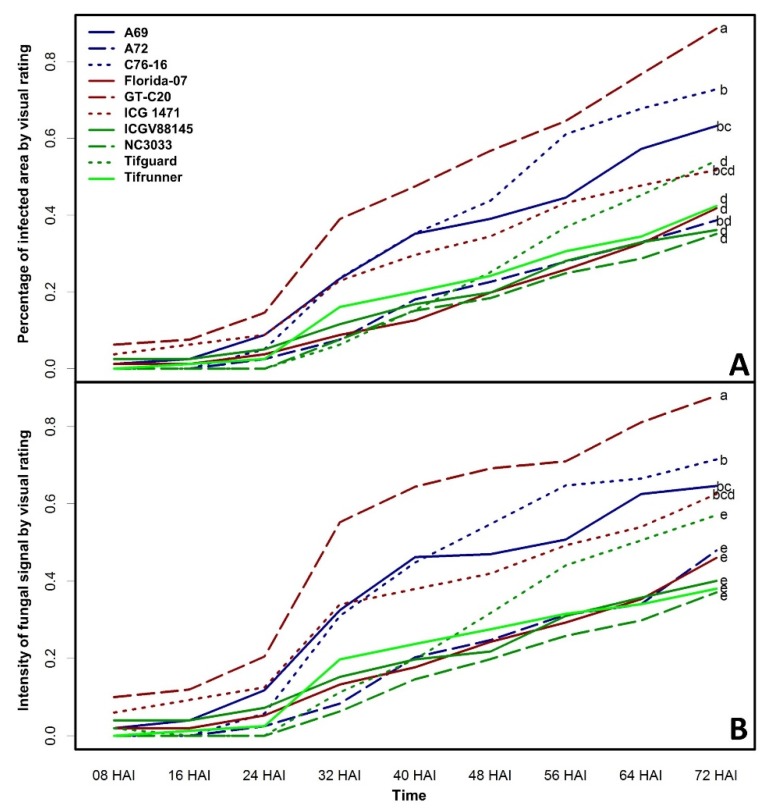
Repeated measure analysis of ten peanut genotypes inoculated with AF-70-GFP strain across nine time points from 8 to 72 h after inoculation (HAI) determined by visual rating. Log-transformed percentage of colonized area (**A**) and fungal GFP intensity (**B**) were presented. Different letters indicate significant differences at *p* < 0.05 level determined by Tukey’s range test.

The AF-70-GFP strain does not differ from wild-type *A. flavus* in terms of pathogen aggressiveness and aflatoxin production [2]. The GFP signal produced by AF-70-GFP is a good indicator of fungal growth allowing non-destructive, real-time monitoring of fungal development. In this study, *A. flavus* fungal growth rates were significantly different among the tested peanut genotypes determined by visual ratings of fungal GFP signal on the seed surface (Figure 1). Fungal growth curves based on visual ratings and reported as percentage of infected area (Figure 1A) and intensity of fungal GFP signal (Figure 1B) gave similar patterns, suggesting either parameter can be applied to monitor fungal development on the seed surface. GFP signal was not detected on seed surfaces freshly inoculated with conidial suspension, whereas newly grown hypha and conidia emitted strong GFP signals. Fungal GFP expression became visible across peanut genotypes between 8 and 24 h after inoculation (HAI) and rapidly increased throughout the 72-h time course. Therefore, under current testing conditions, AF-GFP-70 had a short incubation period of 8–16 h prior to initiating a vigorous growth phase. Tukey’s range test showed that GT-C20 is the genotype most conducive to fungal growth followed by C76-16 and A69. NC 3033 had the least fungal growth on seed surfaces; however, it was not significantly different from Tifrunner, Tifguard, A72, ICGV88145 and Florida-07. ANOVA analysis of area under the disease progress curve (AUDPC) of these two parameters gave similar results (Figure S1). 

**Figure 2:**
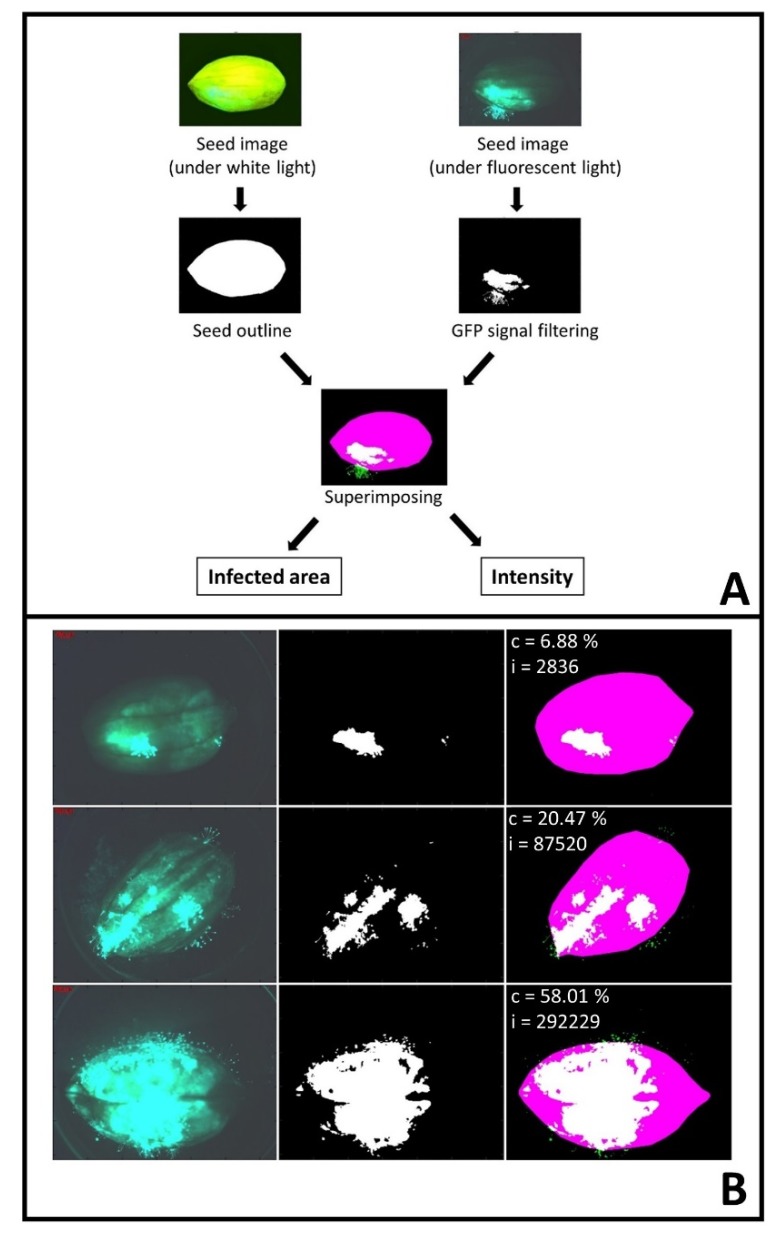
SICIA. (**A**) SICIA data analysis flow chart; (**B**) three examples of SICIA output of percentage of infected area (c) and intensity of GFP signal (i) for seeds with varied levels of fungal infection. Seed sizes are 48.57, 49.02 and 58.94 mm^2^; top, middle and bottom, respectively.

**Figure 3:**
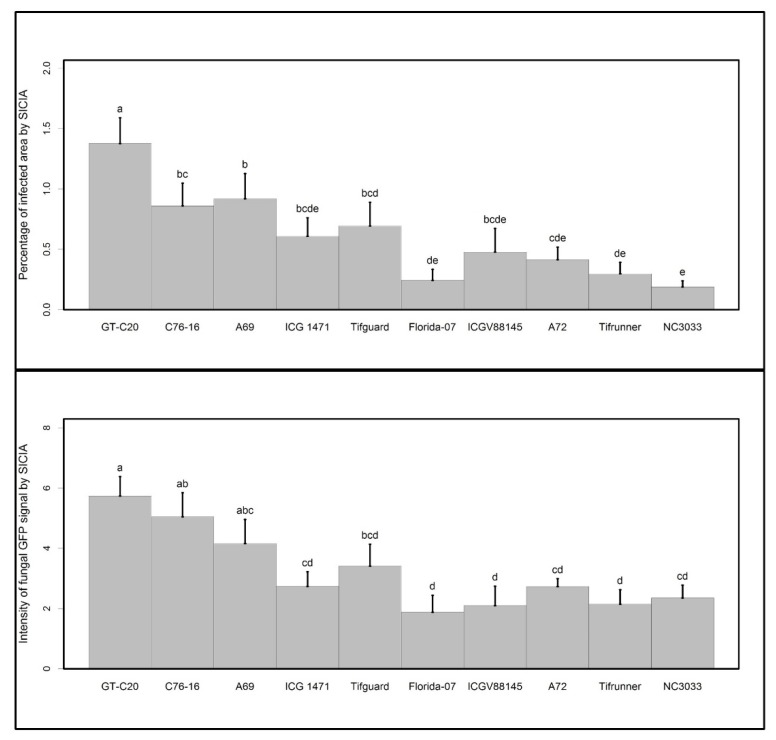
ANOVA analysis of log-transformed values of percentage of the infected area (**upper** panel) and intensity of fungal GFP signal (**lower** panel) determined by SICIA. Different letters indicate significant differences at *p* < 0.05 level determined by Duncan multiple range test.

To objectively determine fungal GFP signal on seed surfaces, seed infection coverage and intensity analyzer (SICIA), a Matlab software, was developed. The software analysis flow chart (Figure 2A) and examples of the fungal infected area and fungal intensity quantification by SICIA (Figure 2B) are provided. Both percentage of infected area, and intensity of fungal GFP signal, estimated by SICIA (Figure 3), confirmed visual rating scores (Figure 1) in that GT-C20, C76-16, and A69 supported the highest level of fungal growth and NC 3033 had the least surface fungal growth. Visual rating across a time course is tedious, which limits the number of genotypes included in a study. On the contrary, image analysis is automated, removes subjectivity, and is fast. In addition, visual rating scores are categorical with limited interpolation, whereas image analysis quantifies GFP signal by continuous measurements, which explains the better separation of means by SICIA than that of visual rating.

**Figure 4:**
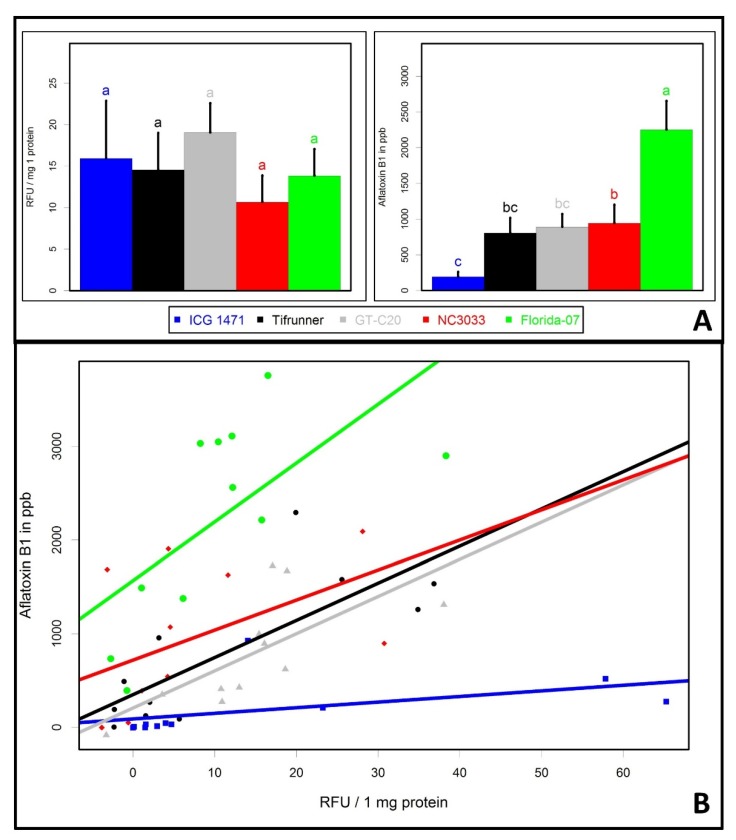
Fungal GFP expression and aflatoxin accumulation in five selected genotypes measured by fluorometric and VICAM assays. (**A**) ANOVA analysis of fungal GFP level, left bar graph, and aflatoxin B level, right bar graph. Different letters indicate significant differences at *p* < 0.05 level determined by Duncan’s multiple range test; (**B**) interaction between GFP and aflatoxin levels, each data point is the average of an experimental block. RFU stands for GFP relative fluorescence unit.

GT-C20, NC 3033, ICG 1471, Tifrunner, and Florida-07 were selected for quantification of GFP and aflatoxin B by fluorometric and VICAM Afla-B column assays (VICAM, Nixa, MO, USA), respectively, since these five genotypes represented the range of fungal growth in this study. GFP and aflatoxin analyses were performed on a single-seed basis for best comparison between aflatoxin production and fungal growth. No statistically significant differences were found for GFP expression among genotypes (Figure 4A). Although NC 3033 appeared to support significantly less fungal growth on the seed surface in the previous analysis, vigorous fungal growth beneath the testa negates the difference on seed surfaces. Previously, tannins extracted from the testa were shown to inhibit *A. flavus* growth [43]. It is possible that testa tannin or phenolic content of NC 3033 may retard fungal growth on the seed surface, which could be of interest for further investigation. In addition, this shows that the progressive fungal growth pattern can be different across peanut genotypes, as it tends to be on the surface in some genotypes and more penetrated inside the seeds of others.

ICG 1471 was found to accumulate significantly less aflatoxin than other genotypes and Florida-07 had the highest level of aflatoxin (Figure 4A). Interaction plots between GFP and aflatoxin level indicated that ICG 1471 produced minimum amounts of aflatoxin regardless of the amount of fungal GFP accumulation (Figure 4B). Florida-07 was the only high oleic genotype [38] among the tested genotypes. Evidence has been reported that high oleic, low linoleic acid ratios in peanut are responsible for an increase in post-harvest aflatoxin accumulation with an in vitro inoculation method [42,45]. However, even among the four normal oleic acid genotypes tested in our study, ICG 1471 accumulated significantly less aflatoxin. 

To further investigate genotypic differences in pre-harvest aflatoxin accumulation, all genotypes were tested in rain exclusion shelters inoculated with *A. flavus* and *A. parasiticus* (Table 1). From the two-year data, ICG 1471 had low aflatoxin production, whereas most tested lines accumulated greater than 20 ppb of aflatoxin. High variation of field aflatoxin accumulation documented here is common for field studies [18], and aflatoxin resistant lines need to be tested for multiple years in multiple environments. Although our in vitro data is consistent with field studies in regard to aflatoxin resistance of ICG 1471, additional field and laboratory studies are needed to define the resistance mechanisms for this genotype. GT-C20 was previously reported to limit *A. flavus* fungal growth and inhibit aflatoxin production [33], which is contrary to our findings. Both our field and in vitro studies demonstrated the high susceptibility of GT-C20 to *A. flavus* fungal growth and aflatoxin production. The seed source of GT-C20 was the same as the male parent of the T-population used for genetic mapping of Tomato Spotted Wilt Virus and leaf spot resistance [46,47]. As mentioned earlier, a truly aflatoxin resistant line should not be claimed unless it withstands multiple tests due to the highly variable nature of the *A. flavus*/peanut host/secondary product biosynthesis interaction.

## 3. Conclusions

Highly reproducible results from this study support the robustness of these methods applied to quantify *A. flavus* growth. Differential genotypic responses to *A. flavus* fungal growth and aflatoxin production were revealed. Peanut germplasm ICG 1471 was found to inhibit aflatoxin production without restraining fungal growth. Rainout shelter testing also showed reduced aflatoxin production in ICG 1471, suggesting that this germplasm and its underlying genetic mechanisms for resistance may be useful in breeding for pre- and post-harvest aflatoxin reduction. To further characterize potential mechanisms of resistance in ICG 1471, RNA-seq analysis carried out in our lab revealed the genetic pathways differentially regulated in this resistant line upon *A. flavus* infection (manuscript in preparation).

## 4. Materials and Methods

### 4.1. Plant Materials

Ten peanut genotypes were planted on the Tifton Campus of the University of Georgia in June and harvested in October 2015. Harvest was according to respective maturity dates as follows: ICG88145, ICG 1471, GT-C20: 115 days; C76-16, A72, A69, Tifguard, NC 3033: 135 days; and Tifrunner, Florida-07: 150 days. Mature seeds were selected from each genotype. Seeds were dried at 30 °C for 7–10 days before being stored at 4 °C for 2 to 3 months prior to the initiation of the experiment. Thirty seeds per genotype were used to determine seed viability by a germination test, and another 150 seeds were used for fungal infection and aflatoxin analysis studies described below. 

### 4.2. In Vitro *A. flavus* Inoculation Using AF-70-GFP

This experiment was conducted with a randomized complete block (RCB) design with 4 blocks consisting of five plates in each block. Each plate hosted 10 seeds from the ten peanut genotypes included in this study and were randomly distributed on the plate. The experiment was replicated three times. One block (5 seeds per genotype) was removed from the study due to contamination from other microorganisms. Therefore, the total number of individual seeds tested in this study was 55 per genotype. Seeds were surface sterilized for 15 min under UV light (LABCONCO purified class II biosafety cabinet, Kansas City, MO, USA). The AF-70-GFP strain [2] was used for infection. This strain had enhanced green fluorescent protein (EGFP) under the control of glyceraldehyde phosphate dehydrogenase (gpdA) promoter and *A. parasiticus* nmt-1 terminator [2]. The fungus was grown on potato dextrose agar (PDA) medium in petri dishes for two weeks at 30 °C, and conidia were suspended in 0.01% Tween 20 solution. Conidia concentration was estimated using a Fuchs-Rosenthal Counting Chamber (Hausser Scientific, Horsham, PA, USA) and was adjusted to 1000 conidia/mL. Twenty milliliters of conidial suspension were used to inoculate every five seeds by immersion for 30 min with quick vortexing every 5 min [48]. A wide range of conidia concentrations (10^4^ to 10^6^ conidia/mL) had been used previously [48,49,50]. A low concentration of conidia was chosen for this study to avoid an overwhelming level of colonization. After inoculation, the seeds were placed in 12-well tissue culture plates (Fisher Scientific, Suwanee, GA, USA) at a density of one seed per well with the two middle wells filled with sterilized water to increase humidity. The plates were sealed and incubated in dark at 30 °C for 72 h.

### 4.3. Visual Tracking of GFP Expression

Fungal growth on the seeds was visually monitored by fungal GFP expression under a microscope (STEMI SV 11 ZEISS equipped with a HBO 100 microscope illumination system, Carl Zeiss Microscopy, Thornwood, NY, USA). GFP signal was observed at 480 ± 30 nm excitation; 545 nm emission wave lengths. Fungal GFP expression on the seed surface was scored visually every eight hours for up to 72 h after inoculation. Percentage of fungal infected area over the surface area of peanut kernels visualized under the microscope was estimated based on a scale of 0 to 5 as follows: 0: no infection to 5%; 1: 5–25%; 2: 25–50%; 3: 50–75%; 4: 75–90%; and 5: >90%. The intensity of fungal GFP signal was estimated based on a scale of 0 to 3 as follows: 0: no infection; 1: low intensity; 2: medium intensity; and 3: high intensity. Initiation of sporulation was estimated by documenting the time point that the first spore became visible on the surface of infected seeds.

### 4.4. Image Processing

At 72 hours, all seeds that had a percentage of infected area higher than 5% were photographed using an Axiocam CCD camera (Carl Zeiss Microscopy, Thornwood, NY, USA). Two images were taken for each seed; one image was taken for GFP visualization and the other image was taken under white light.

Seed Infection Coverage and Intensity Analyzer (SICIA) software was designed with MATLAB R2016a (The University of Georgia campus-wide site licensing agreement, Athens, GA, USA). The software layout is presented (Figure 2A). SICIA extracts the outline of a seed using the image taken under white light. GFP signal was captured from the image taken under fluorescent light and superimposed on to the seed outline to calculate seed size, infected area, GFP signal density, and infection coverage ratio. The program generated a .csv output file containing the calculated values and produced processed images in .jpg format. SICIA is freely available to the public under MIT license and can be downloaded from https://github.com/w-korani/SICIA (Supplemental files: SICIA.exe). SICIA is also calibrated to analyze images from small seeds, e.g., rice, and leaves.

### 4.5. GFP and Aflatoxin Analysis

All seeds were harvested at 72 h after inoculation and ground in liquid nitrogen. A portion of the pulverized tissue was used for total protein extraction in a sucrose-Tris solution (0.5 M and 0.1 M, respectively, pH 7.5 containing 1 mM PMSF) [51]. The tube was then stored on ice for 15 min and centrifuged at 17,530× *g* for 15 min at 4 °C. The supernatant containing extracted protein was quantified by a BCA assay kit (Pierce, Rockford, IL, USA). The GFP relative fluorescence units (RFU) were detected in 100 µL of the protein extract at 485 nm excitation and 535 nm emission wavelengths (Synergy HT Bio-Tek instrument, Bio-Tek Instruments Inc., Winooski, VT, USA). RFU was normalized to 1 mg of the total extracted protein.

Aflatoxin was extracted from a second aliquot of pulverized tissue by adding 200 µL of 25% NaCl and 800 µL of 100% methanol [52]. The tube was vortexed and incubated at room temperature for 30 min. In addition, 100 µL of supernatant was collected after centrifugation at 9000× *g* for 10 min at room temperature, and 400 µL of HPLC-grade water was added. VICAM Afla-B column (VICAM, Nixa, MO, USA) was used to extract aflatoxin B1, and the VICAM Fluorometer (VICAM, Nixa, MO, USA) was used to detect the aflatoxin B concentration according to the manufacturer’s instructions.

### 4.6. Rainout Shelter Study

Pre-harvest susceptibility to aflatoxin accumulation was evaluated in rainout shelters established in Tifton, GA, USA using a randomized block design [53]. The fields were inoculated with *A. flavus* Link ex Fries (NRRL 3357) and *A. parasiticus* (NRRL 2999) at mid-bloom as both fungal strains produce aflatoxin B1, the highly regulated toxin for animal consumption [11]. Drought stress was imposed by moving the rainout shelters over test plots 40 d before harvest. Peanuts from each plot were shelled using a Penco peanut sheller (Peerless Engineering Company, Chula, GA, USA) and ground in a household food processor for about 1 min. Aflatoxin concentration was measured on a 100-g subsample using the VICAM columns as described above.

### 4.7. Statistical Analysis

Statistical analysis was carried out by R3.2.2 using ‘stats’ package. Agricolae package in R3.2.2 [54] was used to perform Duncan’s multiple range test and to calculate the area under the disease progress curve (AUDPC).

## Figures and Tables

**Table 1:** Aflatoxin levels tested in the rainout shelter.

Genotype	Year 2014		Year 2015	
	Aflatoxin Range (ppb)	Average Aflatoxin B (ppb)	Aflatoxin Range (ppb)	Average Aflatoxin B (ppb)
ICG 1471	5 to 35	15	2 to 7	3
Florida-07	5 to 18	11	23 to 641	214
Tifrunner	9 to 48	26	4 to 1200	381
A69	3 to 230	61	3 to 54	20
A72	3 to 679	216	3 to 2100	425
ICGV88145	1 to 1034	226	1 to 11	4
C76-16	25 to 535	246	22 to 599	200
Tifguard	12 to 734	272	n/a	n/a
NC 3033	220 to 360	303	n/a	n/a
GT-C20	220 to 39,000	4581	n/a	n/a

## Supplementary Materials

The following are available online at www.mdpi.com/2072-6651/9/7/218/s1, Figure S1: ANOVA analysis of log-transformed AUDPC values of the ten inoculated peanut genotypes, File: SICIA.exe.

## Abbreviations

The following abbreviations are used in this manuscript.

*A. flavus*	*Aspergillus flavus*
*A. parasiticus*	*Aspergillus parasiticus*
AUDPC	Area Under the Disease Progress Curve
GFP	Green Fluorescent Protein
RFU	Relative Fluorescence Unit
SICIA	Seed Infection Coverage and Intensity Analyzer
