# Supplementary material for: Genotypic Regulation of Aflatoxin Accumulation but Not Aspergillus Fungal Growth upon Post-Harvest Infection of Peanut (Arachis hypogaea L.) Seeds
Source: Toxins (Basel). 2017 Jul 12;9(7):218. doi: 10.3390/toxins9070218 (PMC5535165; doi:10.3390/toxins9070218)
Supplement: Supplementary file 1 [file toxins-09-00218-s001.zip › toxins-197841 supplementary.pdf]

Supplementary Materials: Genotypic Regulation of Aflatoxin Accumulation but not *Aspergillus* Fungal Growth upon Post-Harvest Infection of Peanut (*Arachis hypogaea* L.) Seeds

Walid Ahmed Korani, Ye Chu, Corley Holbrook, Josh Clevenger and Peggy Ozias-Akins

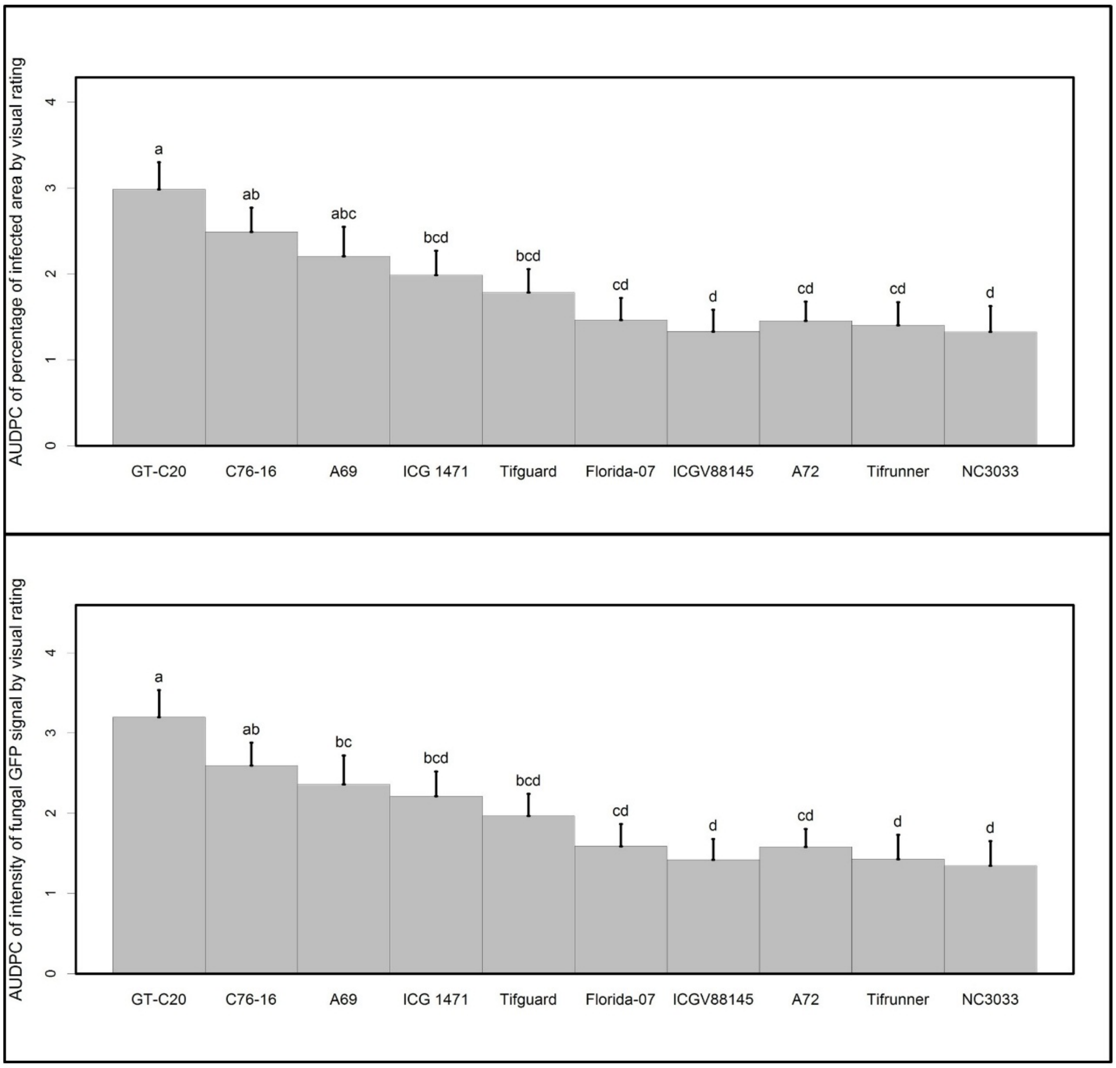

Figure S1: ANOVA analysis of log-transformed AUDPC values of the ten inoculated peanut genotypes. Percentage of infection area (upper panel) and the intensity of the fungal GFP signal (lower panel); Different letters indicate significant differences at $p < 0.05$ level determined by Tukey's range test.
